# Special Issue “Latest Advances in Nanomedicine Strategies for Different Diseases”

**DOI:** 10.3390/ijms25115835

**Published:** 2024-05-27

**Authors:** Nagavendra Kommineni, Veera Ganesh Yerra

**Affiliations:** 1 Center for Biomedical Research (CBR) Population Council, The Rockefeller University, New York, NY 10065, USA; 2 Keenan Research Centre for Biomedical Science, Li Ka Shing Knowledge Institute, St. Michael’s Hospital, Toronto, ON M5B 1W8, Canada; veeraganesh.yerra2@unityhealth.to

We launched this Special Issue amidst the COVID-19 pandemic, spurred by the growing interest in nanotherapeutic formulations for delivering SARS-CoV-2 viral messenger Ribonucleic Acid (mRNA) vaccines [1]. However, our goal extended beyond showcasing nanomedicines solely as viral vaccines; we aimed to present the audience with the latest advancements in nanomedicine, which are poised to revolutionize the treatment landscape for various diseases, including cancer [2]. We are thrilled by the overwhelming response to our Issue, which saw the acceptance of four research articles and two reviews. The articles published in our Special Issue feature advanced and innovative methods for creating novel nanoformulations, addressing significant limitations in delivering nanomedicines to specific targets while minimizing systemic toxicity. Additionally, the review articles in our issue highlight recent advancements in nanotechnology applied to manufacturing mRNA vaccines and developing transition metal-based nanoparticulate systems for cancer research. Overall, through the work presented in our Special Issue, researchers have provided critical insights into optimized nanoformulation strategies for efficient to-brain delivery and enhanced efficiency for treating pancreatic cancer, and into modified nanotheranostics, which aim to provide better tumor-targeting capabilities, as detailed below.

Recent advances in gene therapies have generated immense interest in utilizing gene manipulation strategies to treat several neurological diseases [3]. However, delivering these treatments via the parenteral route creates obstacles to reaching their intended targets and penetrating biological barriers like the blood–brain barrier (BBB) [4]. The BBB, consisting of tight junctions between brain microvascular endothelial cells, acts as a semi-permeable membrane, regulating the entry of foreign substances into the brain. While it effectively blocks toxins, it also hinders the delivery of therapeutics to the brain [4]. Viral vectors like adeno-associated viruses (AAVs), especially AAV9 serotype-based vectors, efficiently bypass the BBB to deliver the cargo to the brain parenchyma. However, they suffer from severe drawbacks, such as immunogenicity and a limited cargo packing capacity [5,6]. Non-viral vectors, such as nanoformulations, overcome these limitations by utilizing modified physiochemical and mechanical properties, stimuli responsiveness, altered size, and surface charge to enclose genetic material efficiently and to enhance the delivery of a wide range of nucleotide-based therapies to the brain [6]. These non-viral vectors include modified liposomal formulations, polymeric nanoparticles, and metal nanoparticles capable of carrying complex biological payloads for treating various neurological diseases [7]. Gold nanoparticles (AuNPs), for instance, are known to enhance BBB permeability by inhibiting the protein kinase c-Zeta (PKC-ζ) isoform, thereby preventing the phosphorylation of Zonula occludens-1 (ZO-1) and occludin, which stabilize the tight junctions in the BBB [8]. Elżbieta Okla et al. harnessed this ability of AuNPs and modified them through Polyethylene Gylcol (PEG)ylation to improve the central nervous system (CNS) delivery of a AuNP/APOE small interfering Ribonucleic Acid (siRNA)formulation for Alzheimer’s disease treatment. Additionally, they showed that these AuNPs/siAPOE4 complexes demonstrated significantly lower cytotoxicity in human brain endothelial cells than free nanoparticles, demonstrating the safety and tolerability of this novel nanotherapeutic formulation [9].

Despite several advancements in cancer chemotherapy, existing therapies often cause dose-related cytotoxicity in unintended organs, leading to severe adverse reactions such as myelosuppression and alopecia [10]. 5-fluorouracil (5-FU), a pyrimidine analogue widely used to treat various cancers such as pancreatic, colorectal, mammary, and skin cancers, faces challenges due to its narrow therapeutic window and variable therapeutic/toxic response among patients, even at the same dose [11]. Despite several attempts to create a nanoformulation of 5-FU to enhance its targetability and selective toxicity, these efforts have yielded limited success in their translations to clinical use [12,13]. As a result, researchers have developed 5-FU analogues by combining it with tetrahydrofuran, resulting in the synthesis of 1,3-bistetrahydrofuran-2yl-5FU (MFU), which has demonstrated high anti-cancer activity in pancreatic cancer cells [14]. In the current Special Issue, N. B. Ndemazie et al. attempted to create lipid-based nanoparticles of MFU, called Zhubech. They evaluated Zhubech’s stability, release kinetics, and in vitro and in vivo activities against pancreatic cancer. Zhubech showed increased cellular uptake and enhanced cytotoxicity in pancreatic cancer cell lines (Miapaca-2 and Panc-1) and a reduced tumor volume in Patient-Derived Xenograft (PDX)-bearing mice, compared to MFU alone [15].

Nanotheranostics offer an ideal combination of therapeutic drugs and diagnostic agents in a single system, facilitating the simultaneous identification and targeting of specific biomarkers. This integration of diagnostic and therapeutic components provides unique opportunities for monitoring drug release, guiding therapy through imaging, patient stratification, and monitoring treatment response [16]. In particular, the advent of novel imaging diagnostics for assessing tumor vascularity and vessel permeability aids in delivering optimal doses of cytotoxic agents to the precise location, thereby minimizing systemic toxicity in cancer therapy [17]. Human serum albumin (HSA) has increasingly been used as a drug carrier due to its biocompatibility. To enhance its magnetic resonance imaging (MRI) contrast capability, it is often combined with CAN-stabilized maghemite ((CeL_n_)^3/4+^-γ-Fe_2_O_3_) to create dual-phase nano systems [18]. Julia Malinovskaya et al. utilized this dual system by conjugating it with Polylactic-co-Gylcolic Acid (PLGA)-based doxorubicin nanoparticles. Both types of nanoparticles accumulated in the same tumor and peritumoral regions, exhibiting the highest co-distribution in mice bearing 4T1-mScarlet murine mammary carcinoma [19]. Hence, this unique combination of HSA–maghemite nanoparticles and PLGA nanoparticles appears to be ideal for theranostic applications. Another challenge in theranostic nanoformulations is limited access to the tumor site. To enhance tumor accessibility and the retention of nanotheranostics, a double-targeting strategy can be employed, interacting with both tumor endothelium and surface receptors on tumor tissue cells [20]. In their study, N. Yabbarov et al. used polyamide amine dendrimers with gadolinium, surface-functionalized with selectin ligands and alpha-fetoprotein receptor ligands. The resulting nanoparticles accumulate in the tumor microenvironment by interacting with P- and E-selectins and are internalized into cancer cells through interactions with alpha-fetoprotein ligands and their corresponding receptors [21]. Thus, this dual-targeting system enhances tumor retention and increases anti-cancer activity compared to mono-targeting systems.

The perspectives of various authors in our Special Issue articles will guide future studies towards developing alternative and improved nanotherapeutics, offering unique and innovative solutions to address future health challenges.

## References

[1] Chung Y.H., Beiss V., Fiering S.N., Steinmetz N.F. (2020). COVID-19 Vaccine Frontrunners and Their Nanotechnology Design. ACS Nano.

[2] Patra J.K., Das G., Fraceto L.F., Campos E.V.R., Rodriguez-Torres M.d.P., Acosta-Torres L.S., Diaz-Torres L.A., Grillo R., Swamy M.K., Sharma S. (2018). Nano based drug delivery systems: Recent developments and future prospects. J. Nanobiotechnol..

[3] Morris J.A., Boshoff C.H., Schor N.F., Wong L.M., Gao G., Davidson B.L. (2021). Next-generation strategies for gene-targeted therapies of central nervous system disorders: A workshop summary. Mol. Ther..

[4] Wu D., Chen Q., Chen X., Han F., Chen Z., Wang Y. (2023). The blood–brain barrier: Structure, regulation, and drug delivery. Signal Transduct. Target. Ther..

[5] Lentz T.B., Gray S.J., Samulski R.J. (2012). Viral vectors for gene delivery to the central nervous system. Neurobiol. Dis..

[6] Xie R., Wang Y., Burger J.C., Li D., Zhu M., Gong S. (2023). Non-viral approaches for gene therapy and therapeutic genome editing across the blood-brain barrier. Med. X.

[7] Nance E., Pun S.H., Saigal R., Sellers D.L. (2022). Drug delivery to the central nervous system. Nat. Rev. Mater..

[8] Li C.H., Shyu M.K., Jhan C., Cheng Y.W., Tsai C.H., Liu C.W., Lee C.C., Chen R.M., Kang J.J. (2015). Gold Nanoparticles Increase Endothelial Paracellular Permeability by Altering Components of Endothelial Tight Junctions, and Increase Blood-Brain Barrier Permeability in Mice. Toxicol. Sci..

[9] Okła E., Białecki P., Kędzierska M., Pędziwiatr-Werbicka E., Miłowska K., Takvor S., Gómez R., de la Mata F.J., Bryszewska M., Ionov M. (2023). Pegylated Gold Nanoparticles Conjugated with siRNA: Complexes Formation and Cytotoxicity. Int. J. Mol. Sci..

[10] Anand U., Dey A., Chandel A.K.S., Sanyal R., Mishra A., Pandey D.K., De Falco V., Upadhyay A., Kandimalla R., Chaudhary A. (2023). Cancer chemotherapy and beyond: Current status, drug candidates, associated risks and progress in targeted therapeutics. Genes. Dis..

[11] Li M., Mindt S., Lück A., Hutzschenreuter U., Kollendt M., Lathan B., Zöller T., Frank-Gleich S., Lorentz C., Lamberti C. (2023). Drug monitoring detects under- and overdosing in patients receiving 5-fluorouracil-containing chemotherapy-results of a prospective, multicenter German observational study. ESMO Open.

[12] Entezar-Almahdi E., Mohammadi-Samani S., Tayebi L., Farjadian F. (2020). Recent Advances in Designing 5-Fluorouracil Delivery Systems: A Stepping Stone in the Safe Treatment of Colorectal Cancer. Int. J. Nanomed..

[13] Nirmala M.J., Kizhuveetil U., Johnson A., Balaji G., Nagarajan R., Muthuvijayan V. (2023). Cancer nanomedicine: A review of nano-therapeutics and challenges ahead. RSC Adv..

[14] Ndemazie N.B., Inkoom A., Ebesoh D., Bulusu R., Frimpong E., Trevino J., Han B., Zhu X., Agyare E. (2022). Synthesis, characterization, and anticancer evaluation of 1,3-bistetrahydrofuran-2yl-5-FU as a potential agent for pancreatic cancer. BMC Cancer.

[15] Ndemazie N.B., Bulusu R., Zhu X.Y., Frimpong E.K., Inkoom A., Okoro J., Ebesoh D., Rogers S., Han B., Agyare E. (2023). Evaluation of Anticancer Activity of Zhubech, a New 5-FU Analog Liposomal Formulation, against Pancreatic Cancer. Int. J. Mol. Sci..

[16] Chen H., Zhang W., Zhu G., Xie J., Chen X. (2017). Rethinking cancer nanotheranostics. Nat. Rev. Mater..

[17] Kashyap B.K., Singh V.V., Solanki M.K., Kumar A., Ruokolainen J., Kesari K.K. (2023). Smart Nanomaterials in Cancer Theranostics: Challenges and Opportunities. ACS Omega.

[18] Israel L.L., Kovalenko E.I., Boyko A.A., Sapozhnikov A.M., Rosenberger I., Kreuter J., Passoni L., Lellouche J.P. (2015). Towards hybrid biocompatible magnetic rHuman serum albumin-based nanoparticles: Use of ultra-small (CeLn)3/4+ cation-doped maghemite nanoparticles as functional shell. Nanotechnology.

[19] Malinovskaya J., Salami R., Valikhov M., Vadekhina V., Semyonkin A., Semkina A., Abakumov M., Harel Y., Levy E., Levin T. (2022). Supermagnetic Human Serum Albumin (HSA) Nanoparticles and PLGA-Based Doxorubicin Nanoformulation: A Duet for Selective Nanotherapy. Int. J. Mol. Sci..

[20] Mashayekhi V., Xenaki K.T., van Bergen En Henegouwen P.M.P., Oliveira S. (2020). Dual Targeting of Endothelial and Cancer Cells Potentiates In Vitro Nanobody-Targeted Photodynamic Therapy. Cancers.

[21] Yabbarov N., Nikolskaya E., Sokol M., Mollaeva M., Chirkina M., Seregina I., Gulyaev M., Pirogov Y., Petrov R. (2022). Synergetic Enhancement of Tumor Double-Targeted MRI Nano-Probe. Int. J. Mol. Sci..

